# Deletion of *Ptpmt1* by *αMHC-Cre* in Mice Results in Left Ventricular Non-Compaction

**DOI:** 10.3390/jdb13030025

**Published:** 2025-07-18

**Authors:** Lei Huang, Maowu Cao, Xiangbin Zhu, Na Li, Can Huang, Kunfu Ouyang, Ze'e Chen

**Affiliations:** Department of Cardiovascular Surgery, Peking University Shenzhen Hospital, School of Chemical Biology and Biotechnology, Peking University Shenzhen Graduate School, Peking University, Shenzhen 518055, China; doctorlay@163.com (L.H.); caomao5wu@163.com (M.C.); izhuxb@163.com (X.Z.); linanana99@163.com (N.L.); huangycan@pkuszh.com (C.H.)

**Keywords:** *Ptpmt1*, mitochondrial phosphatase, left ventricular non-compaction, heart development, mitochondria

## Abstract

*Background:* Left ventricular non-compaction cardiomyopathy (LVNC) is a congenital heart disease characterized by abnormal prenatal development of the left ventricle that has an aberrantly thick trabecular layer and a thinner compacted myocardial layer. However, the underlying molecular mechanisms of LVNC regulated by mitochondrial phosphatase genes remain largely unresolved. *Methods:* We generated a mouse model with cardiac-specific deletion (CKO) of *Ptpmt1*, a type of mitochondrial phosphatase gene, using the *αMHC-Cre*, and investigated the effects of cardiac-specific *Ptpmt1* deficiency on cardiac development. Morphological, histological, and immunofluorescent analyses were conducted in *Ptpmt1* CKO and littermate controls. A transcriptional atlas was identified by RNA sequencing (RNA-seq) analysis. *Results:* We found that CKO mice were born at the Mendelian ratio with normal body weights. However, most of the CKO mice died within 24 h after birth, developing spontaneous ventricular tachycardia. Morphological and histological analysis further revealed that newborn CKO mice developed an LVNC phenotype, evidenced by a thicker trabecular layer and a thinner myocardium layer, when compared with the littermate control. We then examined the embryonic hearts and found that such an LVNC phenotype could also be observed in CKO hearts at E15.5 but not at E13.5. We also performed the EdU incorporation assay and demonstrated that cardiac cell proliferation in both myocardium and trabecular layers was significantly reduced in CKO hearts at E15.5, which is also consistent with the dysregulation of genes associated with heart development and cardiomyocyte proliferation in CKO hearts at the same stage, as revealed by both the transcriptome analysis and the quantitative real-time PCR. Deletion of *Ptpmt1* in mouse cardiomyocytes also induced an increase in phosphorylated eIF2α and ATF4 levels, indicating a mitochondrial stress response in CKO hearts. *Conclusions:* Our results demonstrated that *Ptpmt1* may play an essential role in regulating left ventricular compaction during mouse heart development.

## 1. Introduction

The heart is the first functional organ to develop at embryonic day 7.5 (E7.5), which is responsible for the embryo’s survival with the integrity of structure and function [1]. This intricate process of heart development consists of two key events, including the formation of the myocardium trabecular layer, which meets the oxygen and blood requirements during the embryonic stage, and the myocardium compact layer, which facilitates the formation of a functional ventricular wall [2,3]. Left ventricular non-compaction cardiomyopathy (LVNC), a type of congenital heart disease (CHD), has a higher ratio of morbidity and mortality around the world, resulting from abnormal development of the left ventricle at the embryonic stage [4,5]. In LVNC patients, the left ventricle becomes hypertrabeculated in the trabecular zone and noncompacted in the compact zone during embryonic development [6]. Meanwhile, cardiomyocytes (CMs) in LVNC patients exhibit abnormal regulatory processes, including cardiomyocyte proliferation, cardiomyocyte polarity, and myofibrillogenesis [6,7]. However, the underlying mechanisms of the cause of LVNC are still not fully understood.

Mitochondria constitute nearly 40% of CM content and play multiple critical roles, including cellular homeostasis [8], energetics [9], and metabolism [10]. While LVNC can be caused by various genetic mutations, particularly those affecting sarcomere and cytoskeletal proteins, for example, mutations in the *DES* gene, encoding the intermediate filament protein Desmin, which could also cause LVNC [11,12], mitochondrial dysfunction is a significant factor in its etiology. Growing evidence shows that mitochondrial dysfunction is frequently linked with LVNC. Specifically, mutations in mitochondrial DNA (mtDNA) and resultant defects in complex I, for example, have been associated with LVNC [13,14,15]. LVNC is also frequently observed in individuals with mitochondrial disorders, such as Barth syndrome and Leber hereditary optic neuropathy [14,16]. PTPMT1 (protein tyrosine phosphatase mitochondrial 1), localized to the mitochondria and anchored at the inner mitochondrial membrane, has been identified as a novel reversible phosphatase encoded by nuclear DNA [17,18,19], which plays a critical role in cardiolipin biosynthesis. Cardiolipin is a phospholipid crucial for mitochondrial function and linked to various cardiovascular diseases, including cardiomyopathy and Barth syndrome. Research suggests that defects in cardiolipin, particularly in BTHS, can lead to mitochondrial dysfunction and potentially contribute to the development of LVNC-related cardiomyopathies [16,20]. It remains unclear whether PTPMT1, one of the essential enzymes in the cardiolipin biosynthesis pathway, causes LVNC. To address the contributions of PTPMT1 to LVNC, we crossed the *Ptpmt1* floxed mice [21] with the *αMHC-Cre* transgenic mice that express the Cre recombinase driven by the promoter of myosin heavy chain 6 to generate a conditional cardiac-specific *Ptpmt1* knockout mouse (CKO). Morphological and histological analyses, as well as immunofluorescent analysis, were performed in *Ptpmt1* CKO and control mice. Transcriptional profiles between *Ptpmt1* CKO and the control were determined by RNA sequencing analysis, which was confirmed by quantitative RT-PCR and Western blot assays. Our results revealed that the deletion of *Ptpmt1* by *αMHC-Cre* in CMs resulted in perinatal lethality at P1, as evidenced by a decreased ventricular weight to body weight ratio, premature and irregular heartbeats, spontaneous ventricular tachycardia, a reduced myocardium layer in the left ventricle, and an increased trabecular layer in the left ventricle of CKO mice compared to littermate controls. These results suggest that CKO newborns developed an LVNC phenotype when compared to littermate controls. Additionally, deletion of *Ptpmt1* by *αMHC-Cre* also led to a similar LVNC phenotype in CKO hearts at E15.5 but not at E13.5. Furthermore, we found that cardiomyocyte proliferation in both the trabecular layers and the compact zone was significantly decreased in CKO hearts at E15.5, as shown by the EdU incorporation assay. This is also consistent with the dysregulation of genes related to heart development and cardiomyocyte proliferation in CKO hearts at the same stage, as disclosed by both the RNA sequencing analysis and the quantitative real-time PCR. Interestingly, Western blot analysis displayed that deletion of *Ptpmt1* activated the levels of p-eIF2α and ATF4 expression in mouse hearts at E15.5, possibly as a response to mitochondrial stress. In conclusion, our results demonstrate that *Ptpmt1* may be a key regulator in controlling left ventricular compaction during mouse heart development.

## 2. Materials and Methods

### 2.1. Mice and DNA Analysis

All mouse lines used in the present study have been previously described: *Ptpmt1*-floxed (*Ptpmt1**^f/f^***) alleles [21] and *aMHC-Cre**^+^*** mouse [22]. All used mouse lines had a C57BL/6 J background. The floxed *Ptpmt1* mouse line is characterized by exons 1 and 2 of the mouse *Ptpmt1* gene flanked by two LoxP sites. By crossing with the *αMHC-Cre^+^* transgenic mouse, four genotypes of mice were produced through a two-generation breeding step. Among these, *αMHC-Cre**^+^**Ptpmt1**^f/f^*** mice were considered as CKO mice, whereas the littermates *αMHC-Cre**^−^**Ptpmt1**^f/f^*** and *αMHC-Cre**^−^**Ptpmt1**^f/+^*** were considered as control mice. Genomic DNA was extracted from mouse tails and the embryonic yolk sac as previously described [23]. Polymerase chain reaction analysis was utilized to identify the genotypes of all offspring employing the following gene-specific primers (Supplementary Table S1).

### 2.2. Electrocardiogram

Electrocardiogram (ECG) equipment was connected to LabChart 4.2.3 software. All CKO and control newborns were kept on a constant temperature mat at 37–38 °C and then fixed in an upright position. Subsequently, needle electrodes were attached to the right forelimb and two hind limbs of newborns. Without noise and electric wave disturbances, LabChart 4.2.3 recorded the electrical signals for 5–10 min. Stable signals of recorded signals were kept for at least 1 min and then used for data analysis, including P waves, QRS waves, and T waves.

### 2.3. Morphological, Histological, and Immunofluorescent Analyses

Mice were bred following the typical 12-h light/dark cycle, with noon on the day of the observed vaginal plug marked as E0.5. Embryos were freshly isolated and harvested in ice-cold phosphate-buffered saline (PBS), and image operations were performed using stereo microscopes (Zeiss Stemi 2000-C, ZEISS, Oberkochen, Germany) as previously described [23]. Tissues were fixed overnight in 4% paraformaldehyde (PFA) prepared in PBS. To prepare paraffin sections, tissues were dehydrated in an ethanol gradient and xylene and finally embedded in paraffin. As previously instructed, 6-µm-thickness section slices were harvested and subsequently stained with hematoxylin and eosin (H & E) [24]. Similarly, all stained slices were imaged with stereo microscopes (Zeiss Stemi 2000-C, ZEISS, Oberkochen, Germany). The ventricular area’s thickness was measured and analyzed utilizing ImageJ software. To prepare frozen sections, tissues were soaked in sucrose solutions ranging from 15% to 25%, subsequently embedded in optimal cutting temperature (OCT) compound (Sakura Finetek USA Inc., Torrance, CA, USA), cryosectioned, and finally immunostained as previously described [25]. Then, 6-µm-thickness section slices were collected for EdU proliferation staining with the Click-iT EdU Alexa Fluor 647 Imaging Kit (Cat. C10340, Thermo Fisher Scientific, Carlsbad, CA, USA) and for immunostaining with α-actinin antibody (Cat. A7811, Sigma Chemical Co. St.Louis, MO, USA) to assess cardiomyocyte cytoskeleton. DAPI was employed for the purpose of counterstaining nuclei. All immunostained slices were imaged and analyzed independently in a blinded manner, utilizing the Olympus IX73 confocal microscope and ImageJ software (1.54g).

### 2.4. Quantitative Real-Time PCR Analysis

Hearts were dissected and obtained from CKO and control embryos at E13.5 and E15.5. The total RNA content was extracted from embryonic hearts using TRIzol reagent (Thermo Fisher Scientific, Carlsbad, CA, USA), and cDNA was synthesized using TransScript One-Step cDNA Synthesis SuperMix Kit (Transgen Biotech, Beijing, China). Quantitative real-time PCR was then carried out using TransStart Tip Green qPCR SuperMix (Transgen Biotech, Beijing, China) as per the manufacturer’s guidelines, using the following gene-specific primers (Supplementary Table S2). Each sample was tested at least twice. *Gapdh* was defined as the internal control, and the relative transcript abundance of each gene was subsequently normalized to *Gapdh*. Excel software (16.76-23081101) and GraphPad Prism 9 were used for data analysis and drawing bar graphs, respectively.

### 2.5. Protein Isolation and Western Blot Analysis

Freshly isolated embryonic heart samples were homogenized in lysis buffer (20 mM Tris-HCl, 20 mM NaCl, 0.1 mM EDTA, 1% Triton X-100, 0.5% sodium deoxycholate), which contained an inhibitor cocktail including protease and phosphatase (Cat.B15001, Thermo Fisher Scientific, Carlsbad, CA, USA). The protein concentration was measured using the BCA Protein Assay Kit (Cat.23227, Thermo Fisher Scientific, Carlsbad, CA, USA), and standard procedures were used to run SDS-PAGE gels and subsequently transfer them to PVDF membranes (Millipore, St. Louis, MO, USA) as previously described [26]. Each protein was adjusted to have an equal loading content. The primary antibodies are composed of eIF2α (Cat. 9722, Cell Signaling Technologies, Danvers, MA, USA), Phospho-eIF2α (Cat.9721, Cell Signaling Technologies, Danvers, MA, USA), ATF4 (Cat.11815, Cell Signaling Technologies, Danvers, MA, USA), and GAPDH (Cat.Sc-32233, Santa Cruz Biotechnology, Santa Cruz, CA, USA) in the present study. The secondary antibody utilized in this study is HRP-linked anti-rabbit IgG1 (Cat.7074, Cell Signaling Technologies, Danvers, MA, USA).

### 2.6. RNA Sequencing Data Analysis

Embryonic hearts were freshly isolated and sent to Vazyme company for sequencing treatment in a frozen condition. RNA-seq data were analyzed as described in previous studies [27,28]. Briefly, Cutadapt (v1.9.1) was employed to filter sequencing data, and software Hisat2 (v2.0.1) was used for mapping against the Mus musculus genome (GRCm38/mm10). Subsequently, HTSeq (v0.6.1) was utilized to assess gene expression levels. Finally, differentially expressed genes were analyzed by the DESeq2 Bioconductor package. Software (DESeq2 v1.40+) tools, including Perseus and DAVID v6.8, which were applied for bioinformatic analysis.

### 2.7. Statistics

*P*-values were measured using a two-tailed, unpaired Student’s *t*-test in GraphPad Prism 9.0 software. A *p*-value of less than 0.05 was regarded as statistically significant. Survival data were determined using Kaplan–Meier curves and the log-rank test method. All data represent mean ± SD (error bars).

### 2.8. Ethics Statement

All animal care and experiments were performed in line with the guidelines established by the Animal Care and Use Committee (IACUC) at Peking University Shenzhen Graduate School (Shenzhen, China) and approved by the IACUC (Approval #: AP0017). Circumstances with a 12-h day/night cycle and a temperature maintained at 25 °C were strictly administered to animals to support the standard feeding procedures. Procedures were periodically reviewed, and amendments were made as necessary. For qRT-PCR, Western blots, electrocardiogram, and histology-related analyses, we used embryonic ventricular tissues from both male and female embryos comparably. For RNA-seq analysis, we used the embryonic ventricular tissues only from male embryos.

## 3. Results

### 3.1. Constructing a Conditional Cardiomyocyte-Specific Knockout of the Ptpmt1 Mouse Model

**Figure 1:**
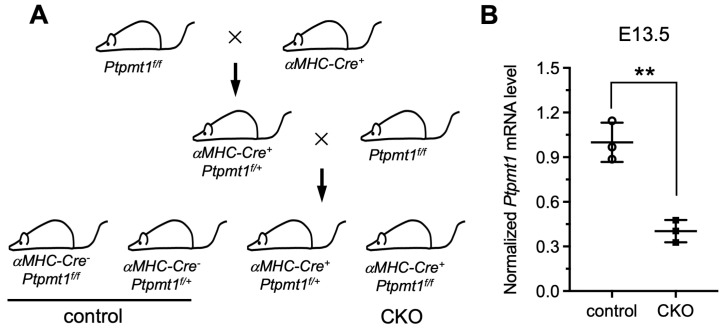
**Generation of a mouse model with a conditional cardiomyocyte-specific knockout of the *Ptpmt1* gene allele**. (**A**) Diagram showing the mouse breeding approach utilized to create α*MHC-Cre^+^Ptpmt1^f/f^* (CKO) mice. The littermates α*MHC-Cre^−^Ptpmt1^f/f^* and α*MHC-Cre^−^Ptpmt1^f/+^* mice were considered as controls. (**B**) qRT-PCR was used to test the knockout efficiency of *Ptpmt1* in cardiomyocytes at E13.5. Data were normalized to the corresponding *Gapdh* levels. *n* = 3 for each group. All data represent mean ± SD. *P*-values were measured by a two-tailed, unpaired Student’s *t*-test. ** *p* < 0.01 versus control.

To date, previous functional studies of *Ptpmt1* have mainly focused on other cell systems, such as its essential role in cardiolipin biosynthesis in mouse embryonic fibroblasts (MEFs) in vitro [21], its regulation of proliferation and glucose metabolism in erythroleukemia cells [29], its promotion of cancer cell death [30], and its mutant disruption of cardiolipin metabolism leading to a neurodevelopmental syndrome [31]. However, the comprehensive physiological function of *Ptpmt1* in cardiomyocytes, especially during heart development in vivo, remains largely obscure. To overcome this challenge, the floxed *Ptpmt1* mouse line was obtained from the Jack E. Dixon laboratory [21]. These mice were born without any observed defects at expected Mendelian ratios, as previously described [21]. To create a cardiomyocyte-specific knockout of the *Ptpmt1* mouse model (CKO), we crossed the floxed *Ptpmt1* mouse with conditional *αMHC-Cre* transgenic mice (Figure 1A). All offspring were born at expected Mendelian ratios. Among them, *αMHC-Cre**^+^**Ptpmt1**^f/f^*** mice were considered as CKO mice at any time in this study, whereas the littermates *αMHC-Cre**^−^**Ptpmt1**^f/f^*** and *αMHC-Cre**^−^**Ptpmt1**^f/+^*** were considered as control mice (Figure 1A). To obtain the above genotypic results, mouse tails and yolk sac tissues were collected for PCR with specific *αMHC-Cre* and *Ptpmt1* primers, respectively. To determine the knockout efficiency of the *Ptpmt1* gene, we checked the expression of the *Ptpmt1* gene between two groups of hearts using specific *Ptpmt1* RT-PCR primers at E13.5. We found a significant decrease of 60% in *Ptpmt1* mRNA levels in CKO hearts compared to littermate controls (Figure 1B), implying a high knockout efficiency in embryonic hearts. These results demonstrate that a conditional cardiomyocyte-specific knockout of *Ptpmt1* mouse model could be a powerful tool for examining the function of *Ptpmt1* in CMs in vivo.

### 3.2. Deletion of Ptpmt1 in Cardiomyocytes Results in Perinatal Lethality

**Figure 2:**
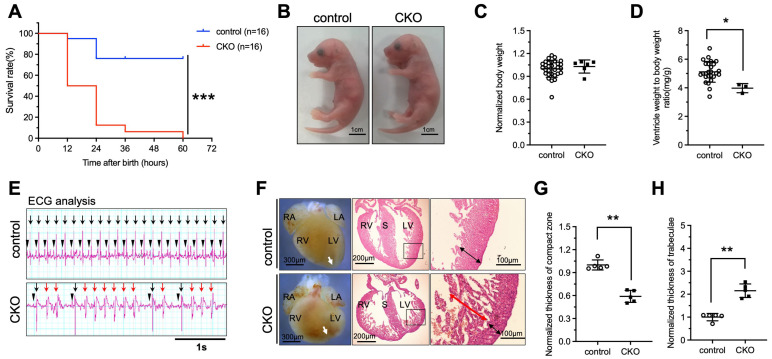
**Deletion of *Ptpmt1* by *αMHC-Cre* in cardiomyocytes results in perinatal lethality**. (**A**) Kaplan–Meier survival curves of *Ptpmt1* CKO (*n* = 16) and control (*n* =16) male mice. *** *p* < 0.001 versus control. (**B**) Representative morphology of CKO and control at postnatal day 1. Scale bar, 1 cm. *n* = 6 for each group. (**C**,**D**) Body weight (mg) (**C**) and ventricle weight to body weight ratio (mg/g) (**D**) analysis of CKO and control at postnatal day 1 (P1), respectively. *n* = 3 at least for each group. All data represent mean ± SD. *p*-values were measured by a two-tailed, unpaired Student’s *t*-test. *p* > 0.05 versus control represents no significance. * *p* < 0.05 versus control. (**E**) Electrocardiogram (ECG) equipment records the electrical signals for newborns at (P1). *n* = 6 for each group. All data represent mean ± SD. *P*-values were measured by a two-tailed, unpaired Student’s *t*-test. Long black arrows indicate normal QRS waves. Long red arrows indicate abnormal QRS waves. Short black arrows indicate normal *p* waves. (**F**) Morphological and histological analyses of control and CKO hearts at P1, respectively. Boxed areas are enlarged on the right. Black arrows represent the compact zone in the left ventricle. Red arrows indicate the trabecular layer in the left ventricle. The white arrows indicate the apex of the heart. Scale bars indicate 300 µm, 200 µm, and 100 µm from left to right, respectively. RA, right atrium; LA, left atrium; RV, right ventricle; LV, left ventricle; S, septum. (**G**,**H**) Quantitative analysis of the thickness of the compact zone (**G**) and the thickness of the trabecular layer (**H**) in the left ventricle at P1. *n* = 5 for each group. All data represent mean ± SD. *P*-values were measured by a two-tailed, unpaired Student’s *t*-test. ** *p* < 0.01 versus control.

A study has demonstrated that global deletion of *Ptpmt1* in mice resulted in embryonic lethality, indicating an essential role of PTPMT1 in the developing stage [21]. To investigate whether deletion of *Ptpmt1* in CMs affects neonatal growth and embryo heart development in both CKO and control, we obtained sufficient offspring and analyzed the survival of CKO and control. Our results revealed that approximately 12.5% of CKO newborns were dead at P2–P3, while approximately 87.5% of CKO newborns were dead at P1 (Figure 2A and Table 1) and showed a significant difference in survival rate when compared with littermate controls (Figure 2A and Table 1), suggesting that the ablation of *Ptpmt1* severely impacts the survival of postnatal mice. To confirm the cause of newborn lethality, we analyzed the whole morphology of both CKO and control newborns. No dramatic difference in gross body morphology was observed between the two groups of newborns (Figure 2B). Statistically, CKO newborns showed a dramatic decrease in the ratio of ventricle weight to body weight compared to littermate controls (Figure 2D), but there was no significant difference in body weight between CKO and littermate controls (Figure 2C), suggesting that the ventricles of the newborns may become defective due to the lack of *Ptpmt1*. Given the essential role of heartbeats in indicating the normal structure of the heart, it is possible that the abnormal hearts showed an irregular heartbeat to some extent. To determine this possibility, we utilized an electrocardiogram (ECG) to assess the heartbeat of newborns. ECG results revealed that CKO newborns had premature and irregular heartbeats, a lower beat frequency, and spontaneous ventricular tachycardia compared to littermate controls (Figure 2E). These results indicate that heart structural defects may occur after *Ptpmt1* ablation. Therefore, we sought to examine the heart structure of the newborns. Morphological analysis uncovered a dramatic change in gross morphology in CKO hearts compared to littermate controls, including an unobvious cardiac apex, decreased long axis, and elongated short axis (Figure 2F). Histological analysis also unraveled a significant 40% decrease in the thickness of the compact zone in the left ventricle of CKO hearts compared to littermate controls (Figure 2F,G). Additionally, there was a one-fold increase in the thickness of trabeculae in the left ventricle of CKO hearts compared to littermate controls (Figure 2F,H). Together, these results are consistent with the LVNC phenotype [32]. These results strongly suggest that *Ptpmt1* is required for maintaining left ventricular compaction in newborn mice.

### 3.3. Loss of Ptpmt1 in Cardiomyocytes Affects Ventricular Wall Development

**Figure 3:**
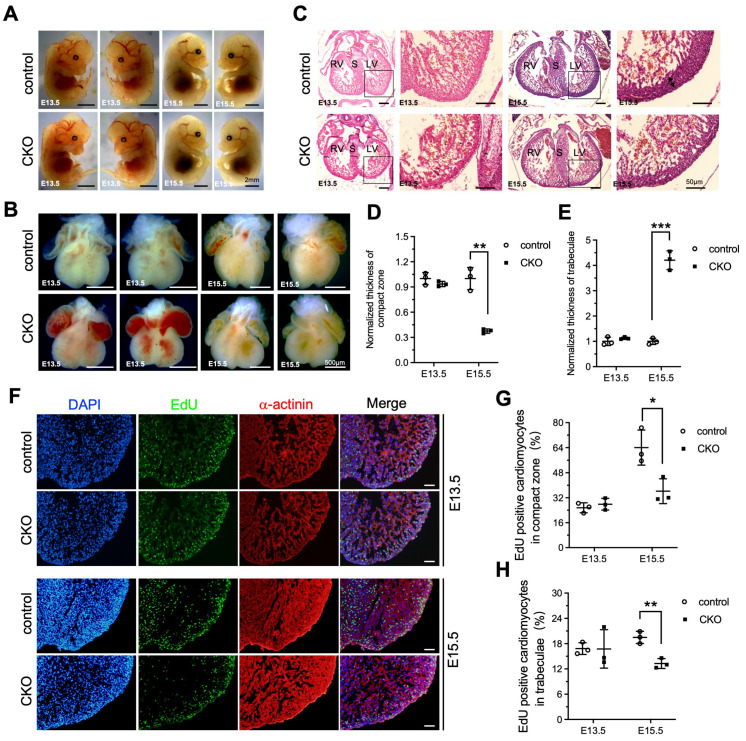
**Loss of *Ptpmt1* in cardiomyocytes affects ventricular wall development**. (**A**) Representative morphology of CKO embryos and littermate controls at both E13.5 and E15.5, respectively. Scale bar, 2 mm. (**B**) Morphological analysis of control hearts and littermate CKOs at both E13.5 and E15.5, respectively. Scale bar, 500 µm. (**C**) Histological analysis of control hearts and littermate CKOs at both E13.5 and E15.5, respectively. Boxed areas are enlarged on the right. Black arrows represent the compact zone in the left ventricle. Scale bar, 50 µm. RV, right ventricle; LV, left ventricle; S, septum. (**D**,**E**) Quantitative analysis of the thickness of the compact zone (**D**) and the thickness of the trabecular layer (**E**) in the left ventricle at E13.5 and E15.5. *n* = 3 for each group. (**F**) Representative immunostaining images of EdU-labeled (green) heart sections from *Ptpmt1* CKO and control mice at E13.5 and E15.5, using an antibody against α-actinin as a cardiomyocyte marker (red). Nuclei is stained with DAPI (blue). Scale bar, 100 µm. (**G**,**H**) Quantification (%) of EdU-positive cardiomyocytes (CM) in the compact zone (**G**) and trabecular layer (**H**) at E13.5 and E15.5. *n* = 3 for each group. All data represent mean ± SD. *p*-values were measured by a two-tailed, unpaired Student’s *t*-test. *p* > 0.05 versus control represents no significance. * p < 0.05 versus control. ** p < 0.01 versus control. *** p < 0.001 versus control.

To investigate whether the LVNC phenotype was appeared at the embryonic stages as a result of *Ptpmt1* loss, we dissected the embryo and the heart from E13.5 to E15.5 to check for changes in both the embryo and heart structure. At E13.5, there were no differences, including gross embryo morphology (Figure 3A), gross heart morphology (Figure 3B), the thickness of the compact zone in the left ventricle (Figure 3C,D), the thickness of trabeculae in the left ventricle (Figure 3C,E), and EdU positive CMs in both the compact zone and trabeculae (Figure 3F–H), as examined in CKO embryos and littermate controls by morphological, histological analyses, and EdU incorporation assay. This demonstrates that deletion of *Ptpmt1* did not affect heart development at E13.5. However, by E15.5, severe left ventricular compaction defects were evident in CKO hearts, such as an abnormal gross heart (Figure 3B), reduced thickness of the compact zone in the left ventricle (Figure 3C,D), and increased thickness of trabeculae in the left ventricle (Figure 3C,E), which is similar to the LVNC phenotype at P1. Although gross embryo morphology did not exhibit any alterations at this stage (Figure 3A). We also found that EdU-positive CMs in the compact zone and trabeculae were significantly reduced in CKO hearts at E15.5 (Figure 3F,G), which may account for the occurrence of LVNC. Taken together, these results suggest that deletion of *Ptpmt1* severely affected left ventricular compaction at E15.5.

### 3.4. Absence of Ptpmt1 in Developing Cardiomyocytes Dysregulates the Transcriptional Profile

**Figure 4:**
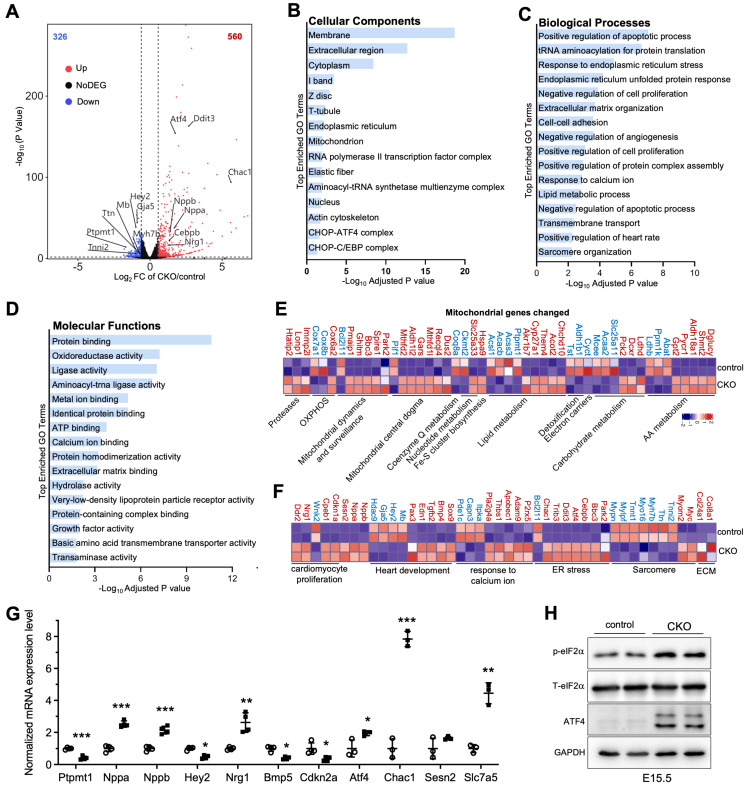
**Transcriptome analysis in CKO and control hearts.** (**A**) Volcano plot analysis of gene expression in CKO versus control hearts at E15.5. Differentially expressed genes (DEGs), including 560 significantly upregulated (red circles) and 326 significantly downregulated (blue circles), were identified in CKO hearts. Adjusted *p* < 0.05 and |log_2_ fold change| > 0.585 were considered as the limit. *n* = 2. (**B**–**D**) Gene Ontology (GO) analysis of DEGs in CKO hearts at E15.5. (**E**,**F**) Heatmap analysis of the DEGs linked to mitochondria, heart development, cell proliferation, ECM, sarcomere, and ER stress in CKO versus control hearts at E15.5. Color bar represents relative expression levels. *n* = 2. (**G**) qRT-PCR validation of representative cardiac genes from RNA-seq data in CKO (circle) versus control (square) hearts at E15.5. *n* = 3-4. Data were normalized to their respective *Gapdh* levels. All data represent mean ± SD. *P*-values were measured by a two-tailed, unpaired Student’s *t*-test. * p < 0.05, ** p < 0.01, and *** p < 0.001 versus control. (**H**) Western blot analysis of the levels of p-eIF2α, T-eIF2α, ATF4, and GAPDH in CKO hearts and littermate controls at E15.5. GAPDH is used as the internal control. *n* = 2.

Based on the above-mentioned findings, we aimed to identify the gene changes associated with ventricular wall development in developing hearts after deletion of *Ptpmt1*. We freshly isolated embryo hearts in CKO and littermate controls at E15.5 for RNA-sequencing assay. From transcriptomic analysis, 886 differentially expressed genes (DEGs), including 560 significantly upregulated and 326 significantly downregulated genes, were identified in *Ptpmt1* CKO hearts compared to littermate controls at E15.5 (Figure 4A). To determine the affected pathways, we used Gene Ontology (GO) analysis to cluster DEGs. As a result, multiple important molecular pathways associated with cardiac development in CKO hearts were enriched, including “positive regulation of heart rate”, “cell proliferation”, “sarcomere organization”, “endoplasmic reticulum stress”, “response to calcium ion”, “extracellular matrix organization”, and “cell-cell adhesion” (Figure 4B–D). Out of 886 DEGs, 49 were mitochondrial genes in CKO hearts (Figure 4E), likely suggesting that mitochondrial homeostasis in gene levels was disturbed in CKO cardiomyocytes due to the ablation of *Ptpmt1*. These results demonstrate that deletion of *Ptpmt1* had apparently affected the cardiac transcriptome atlas.

We next analyzed the molecular changes. Using volcano plot and heatmap analyses, we found that cardiac fetal gene markers atrial natriuretic factor (*Nppa*) and B-type natriuretic peptide (*Nppb*) were significantly upregulated in the hearts of CKO mice at E15.5 (Figure 4A,F). Our qRT-PCR analysis further confirmed a significant decrease in RNA levels of *Nppa* and *Nppb* in CKO hearts, compared to those in control hearts (Figure 4G). These results were in line with the molecular changes in cardiac stress during development [33]. Moreover, upregulated genes were identified in biological pathways associated with “extracellular matrix organization” and “cell-cell adhesion” (Figure 4C,G), indicating the initiation of remodeling and fibrosis processes in hearts of CKO mice to some extent. As expected, some genes, such as *Tnni2*, *Tnnt1*, *Ttn*, *Myh7b*, *and Gaj5,* showed decreased expression levels in response to the observed CKO phenotypes at E15.5 (Figure 4A,F). These genes encode proteins that are essential for cardiomyocyte structure and contractility [34,35,36,37,38]. Furthermore, *Hey2*, which was also markedly downregulated (Figure 4A,F), is crucial for left ventricular maturation [27,39]. Our qRT-PCR result solidly validated the levels of this key heart development gene in cardiac tissue dissected from CKO and littermate controls at E15.5 (Figure 4G). Notably, *Nrg1* exhibited increased expression in CKO hearts (Figure 4A,F), as evidenced by qRT-PCR analysis (Figure 4G), consistent with *Nrg1* regulating trabecular myocardium development [40]. Some critical genes that negatively regulate cardiomyocyte proliferation, such as *Cdkn1a*, *Cpeb1,* and *Ddr2,* were significantly upregulated, but *Wnk2* and *Cdkn2a*, which are related to the promotion of cardiomyocyte proliferation, were dramatically downregulated. These key genes contributed to a significant reduction in cardiomyocyte proliferation in CKO hearts compared to littermate controls (Figure 3F–H). Intriguingly, *Atf4, Cebpb, Ddit3*, *Trib3*, and *Chac1* were coordinately and significantly elevated in CKO hearts (Figure 4A,F,G). This increasing trend in protein expression not only enables *Atf4* to regulate the induction of *Ddit3*/*Chop* in response to endoplasmic reticulum (ER) stress [41] but also may activate the transcription of *Trib3* and *Chac1* in CKO cardiomyocytes, leading to ER stress-induced cardiomyocyte apoptosis, similar to the observations in neuronal contexts [42]. In concert with the increasing trend in *Atf4* expression, the protein levels of ATF4 were remarkably upregulated (Figure 4H). Functionally, eIF2α, which is phosphorylated by both GCN2 and PERK [43,44], is an upstream factor of ATF4. Notably, the phosphorylation level of eIF2α was increased in CKO hearts (Figure 4H), implying that the activation of the eIF2α/ATF4 pathway might be a consequence of mitochondrial stress caused by the deletion of *Ptpmt1.* These results demonstrate that *Ptpmt1* may be an upstream regulator involved in heart development, CM proliferation, and mitochondrial stress, controlling left ventricular compaction development.

## 4. Discussion

In our present study, using a cardiac-specific knockout of the *Ptpmt1* mouse model, we found that CKO newborns had perinatal lethality at P1 compared to littermate controls, as evidenced by the development of a LVNC phenotype. Consistent with the LVNC phenotype at P1, CKO embryo hearts had severely defective left ventricular compaction compared to littermate controls at E15.5. Currently, there are limited valuable investigations about *Ptpmt1* regulating embryonic heart development. Although there is a report that *Ptpmt1* performs a protective role for CMs against necroptosis in induced pluripotent stem cell-derived CMs [45], its in vivo function in CMs remains fully unresolved. Therefore, our findings, which show that deletion of *Ptpmt1* by *αMHC-Cre* leads to a LVNC phenotype, could be indispensable experimental proof for investigators to broaden their understanding of the in vivo physiological function of *Ptpmt1* in CMs.

Overall, abnormal ventricular development results in non-compaction [2,6,46]. During heart development, the ventricles undergo two key morphogenetic changes, including the formation of the trabecular layer and compact zone [3,47,48]. Briefly, as trabeculations undergo compaction, the myocardium compacts inward slowly from the epicardium and from the base toward the apex [46,49]. During compaction, proliferative activity stays elevated in the compact myocardium, leading to a gradual decrease in cardiomyocyte proliferation from the compact zone to the trabecular zone [50,51,52]. Our results also confirmed a higher proliferative activity in the compact zone at both E15.5 and E13.5. Yet, no significant difference in cardiomyocyte proliferation was calculated between CKO and control hearts at E13.5. However, by E15.5, cardiomyocyte proliferation in the compact zone and trabecular layer was significantly reduced in CKO hearts compared to littermate controls, which may be a cause of LVNC phenotype. Apart from changes in cellular proliferation levels, the development of myocardium was regulated by key genes related to CM proliferation. For instance, *Cdkn1a*, as a member of the cyclin-dependent kinase inhibitor family, leads to CM cycle arrest [53], while *Cdkn2a* causes cellular hyperproliferation [53]. Our RNA-seq and qRT-PCR results showed that *Cdkn1a* was upregulated in mutant hearts, while *Cdkn2a* was downregulated in mutant hearts. Both of these two genes together induced a reduction in CM proliferation in the compact zone and trabecular layer. Therefore, the key genes dysregulated at the molecular level and the lower proliferative activity in myocardium at the cellular level are the two causes of the LVNC phenotype appearing in CKO hearts at both E15.5 and P1. On the other hand, transcriptome analysis discovered that key genes promoting ventricular wall development, such as *Mb*, *Hey2*, *Gja5*, and *Ttn*, were downregulated, which is consistent with left ventricular non-compaction at P1 and E15.5. This suggests that *Ptpmt1* may positively regulate these genes involved in ventricular wall development, offering insight into the regulation mechanism of mammalian heart development. In fact, the ER stress response is required for cardiac development [54]. However, the involvement of mitochondrial stress in the development of heart disease is not well characterized [55]. Our results showed that genes responding to mitochondrial stress, including *Ddit3*, *Trib3*, and *Chac1*, were also remarkably activated under the deletion of the *Ptpmt1* condition, suggesting that mitochondrial stress may occur and induce cardiomyocyte apoptosis due to the absence of *Ptpmt1*. Importantly, levels of p-eIF2α and ATF4 showed a common increase, indicating that ATF4 was activated by phosphorylated eIF2α [43]. The integrated activation of the eIF2α/ATF4 pathway may be a consequence of mitochondrial stress [56]. This may confirm that deletion of *Ptpmt1* triggered mitochondrial stress in CKO hearts, leading to the occurrence of a LVNC phenotype. In addition, the mechanism of how mitochondrial stress triggers the eIF2α/ATF4 pathway remains largely unknown. Therefore, mitochondrial stress caused by deletion of *Ptpmt1* activates the eIF2α/ATF4 pathway in CKO hearts, providing new evidence for researchers to better understand the relationship between mitochondrial stress and the eIF2α/ATF4 pathway. In this context, it will be worthwhile to investigate the mechanism of how mitochondrial stress regulates the eIF2α/ATF4 pathway in CMs in future studies. In our previous study, we found that *Ptpmt1* is required for embryonic cardiac cardiolipin biosynthesis to regulate mitochondrial morphogenesis and heart development [57]. The deletion of *Ptpmt1* with Troponin T-Cre, which induces early recombination at E7.5 with high efficiency in cardiomyocytes under the control of the rat cardiac troponin T2, resulted in embryonic lethality between E16.5 and E18.5. The previous work mainly focuses on cardiolipin metabolism as well as mitochondrial structure and function. Whereas, in the present study, we mainly focus on the role of *Ptpmt1* in LVNC, which is totally different from our previous work. Hopefully, it would be beneficial to understand the underlying mechanisms of LVNC.

## 5. Study Limitations

The present study has certain constraints. After the ablation of *Ptpmt1*, we only focus on the altered phenotypes of the left ventricle during embryonic heart development, even at P1. According to the morphological and histological results of control and CKO hearts at P1, we cannot exclude the possibility that Arrhythmogenic right ventricular cardiomyopathy (ARVC) [58] may appear under the condition of LVNC development. Additionally, cardiac inflammation is frequently observed in murine cardiomyopathy, leading to cardiomyocyte death and tissue remodeling [59]. We examined cell death in the heart sections of control and Ptpmt1 knockout embryos at the stage E15.5 with TUNEL staining. However, we could not observe any differences. We did not check for cardiac inflammation. In the future, we will investigate these two significant aspects.

## 6. Conclusions

In summary, these strong pieces of evidence emphasized that *Ptpmt1* may play an essential role in the pathways associated with heart development, cardiomyocyte proliferation, and mitochondrial stress, thus governing the development of left ventricular compaction during mouse heart development. Our findings also provide strong complementary evidence for investigating the indispensable and regulatory role of *Ptpmt1* in left ventricular compaction.

## Figures and Tables

**Table 1:** Genotypic analysis of embryos and pups from *αMHC-Cre^+^ Ptpmt1^f/+^* male with *Ptpmt1^f/f^* female intercrosses.

	Genotypes				
Developmental Stages	*αMHC-Cre^−^ Ptpmt1^f/+^*	*αMHC-Cre^−^ Ptpmt1^f/f^*	*αMHC-Cre^+^ Ptpmt1^f/+^*	*αMHC-Cre^+^ Ptpmt1f/f*	Total
E13.5	27 (25.96%)	25 (24.03%)	24 (23.07%)	28 (26.92%)	104
E15.5	45 (24.47%)	44 (25.88%)	45 (26.47%)	36 (21.17%)	170
P1 ^a^	75 (24.83%)	83 (27.57%)	83 (27.5%)	61 (20.19%) ^b^	302
P10	61 (34.6%)	56 (31.8%)	59 (33.5%)	0 (0.0%)	176

^a^ P1, Postnatal day 1. ^b^ 53 of 61 newborns were dead.

## Data Availability

Data are available in a publicly accessible repository.

## Supplementary Materials

The following supporting information can be downloaded at https://www.mdpi.com/article/10.3390/jdb13030025/s1. Supplementary Table S1: Polymerase chain reaction (PCR) primers; Supplementary Table S2: Quantitative Real-Time PCR primers.
