# Supplementary material for: Deletion of Ptpmt1 by αMHC-Cre in Mice Results in Left Ventricular Non-Compaction
Source: J Dev Biol. 2025 Jul 18;13(3):25. doi: 10.3390/jdb13030025 (PMC12286240; doi:10.3390/jdb13030025)
Supplement: Supplementary file 1 [file jdb-13-00025-s001.zip › Supplementary Table S1 Polymerase chain reaction (PCR) primers..pdf]

Supplementary Table S1: Polymerase chain reaction (PCR) primers.

Gene names	Forward (5′→3′)	Reverse (5′→3′)
aMHCCre	GCCATAGGCTACGGTTAAAAG	ATAATCGCGAACATCTTCAGGT
Ptpmt1	ACTATGAACGAGGAGTACGAGACC	GACTGA-CCCAGAGCTCACTACATA
