# Supplementary material for: Deletion of Ptpmt1 by αMHC-Cre in Mice Results in Left Ventricular Non-Compaction
Source: J Dev Biol. 2025 Jul 18;13(3):25. doi: 10.3390/jdb13030025 (PMC12286240; doi:10.3390/jdb13030025)
Supplement: Supplementary file 1 [file jdb-13-00025-s001.zip › Supplementary Table S2 Quantitative Real-Time PCR primers..pdf]

Supplementary Table S2: Quantitative Real-Time PCR primers.

Gene names	Forward (5′→3′)	Reverse (5′→3′)
Ptpmt1	CGCTGCTCTACACAGTGTTG	GGGTCTCGTACTCCTCGTTC
Gapdh	TGGCCTTCCGTGTTCCTAC	GAGTTGCTGTTGAAGTCGCA
Atf4	GCCCAAACCTTATGACCCAC	TAGCTCCTTACACTCGCCAG
Chac1	TTGCCTATAGTGACAGCCGT	GCTCCCCTCGAACTTGGTAT
Sesn2	ATAACACCATCGCCATGCAC	GGGTCGTCTTCTCAGGGTAG
Slc7a5	CTTAGTCACGTGTCCTGGGT	CTGTGGACACCTGACGTTTG
Nppa	TCGTCTTGGCCTTTTGGCT	TCCAGGTGGTCTAGCAGGTTCT
Nppb	AAGCTGCTGGAGCTGATAAGA	GTTACAGCCCAAACGACTGAC
Hey2	AAGCGCCCTTGTGAGGAAAC	GGTAGTTGTCGGTGAATTGGAC
Nrg1	ATGGAGATTTATCCCCCAGACA	GTTGAGGCACCCTCTGAGAC
Bmp5	TTACTTAGGGGTATTGTGGGCT	CCGTCTCTCATGGTTCCGTAG
Cdkn2a	CTGGAAGAAGTCTGCGTCGG	GTCTTGCCAAAGCGGTTCAG
